# Different genes may be involved in distal and local sensitization: A genome‐wide gene‐based association study and meta‐analysis

**DOI:** 10.1002/ejp.1902

**Published:** 2022-01-07

**Authors:** Afroditi Kouraki, Michael Doherty, Gwen S. Fernandes, Weiya Zhang, David A. Walsh, Anthony Kelly, Ana M. Valdes

**Affiliations:** ^1^ Academic Rheumatology School of Medicine University of Nottingham Nottingham City Hospital Nottingham UK; ^2^ NIHR Nottingham Biomedical Research Centre University of Nottingham Nottingham UK; ^3^ Pain Centre Versus Arthritis University of Nottingham Nottingham UK; ^4^ Versus Arthritis Centre for Sports, Exercise and Osteoarthritis University of Nottingham Nottingham UK; ^5^ Population Health Sciences Bristol Medical School University of Bristol Bristol UK

## Abstract

**Background:**

Neuropathic pain symptoms and signs of increased pain sensitization in osteoarthritis (OA) patients may explain persistent pain after total joint replacement (TJR). Therefore, identifying genetic markers associated with pain sensitization and neuropathic‐like pain phenotypes could be clinically important in identifying targets for early intervention.

**Methods:**

We performed a genome‐wide gene‐based association study (GWGAS) using pressure pain detection thresholds (PPTs) from distal pain‐free sites (anterior tibia), a measure of distal sensitization, and from proximal pain‐affected sites (lateral joint line), a measure of local sensitization, in 320 knee OA participants from the Knee Pain and related health in the Community (KPIC) cohort. We next performed gene‐based fixed‐effects meta‐analysis of PPTs and a neuropathic‐like pain phenotype using genome‐wide association study (GWAS) data from KPIC and from an independent cohort of 613 post‐TJR participants, respectively.

**Results:**

The most significant genes associated with distal and local sensitization were *OR5B3* and *BRDT*, respectively. We also found previously identified neuropathic pain‐associated genes—*KCNA1*, *MTOR*, *ADORA1* and *SCN3B*—associated with PPT at the anterior tibia and an inflammatory pain gene—*PTAFR*—associated with PPT at the lateral joint line. Meta‐analysis results of anterior tibia and neuropathic‐like pain phenotypes revealed genes associated with bone morphogenesis, neuro‐inflammation, obesity, type 2 diabetes, cardiovascular disease and cognitive function.

**Conclusions:**

Overall, our results suggest that different biological processes might be involved in distal and local sensitization, and common genetic mechanisms might be implicated in distal sensitization and neuropathic‐like pain. Future studies are needed to replicate these findings.

**Significance:**

To the best of our knowledge, this is the first GWAS for pain sensitization and the first gene‐based meta‐analysis of pain sensitization and neuropathic‐like pain. Higher pain sensitization and neuropathic pain symptoms are associated with persistent pain after surgery hence, identifying genetic biomarkers and molecular pathways associated with these traits is clinically relevant.

## 1 INTRODUCTION

It is estimated that pain is not adequately controlled in 4 of 10 people that suffer from knee osteoarthritis (OA) (Neogi, 2013). Knee pain hugely contributes to disability (Guccione et al., 1994; March & Bagga, 2004). Although OA pain has traditionally been associated with peripheral pain mechanisms due to joint damage and inflammation, the presence of radiographic joint pathology does not always explain the severity of pain (Finan et al., 2013; Valdes et al., 2012). Many people with OA suffer from pain that they describe as numbness, electric shocks, or a burning, itching sensation that can initiate spontaneously and in the absence of a painful stimulus, suggestive of a neuropathic source (den Boer et al., 2019; Cavalli et al., 2019). A role of the central nervous system (CNS) in augmenting nociceptive processing has been described in OA, which is known as pain sensitization and displays similar characteristics to neuropathic pain (Chappell et al., 2009; Petersen et al., 2015; Soni et al., 2019).

Quantitative sensory testing (QST) can be used to quantify alterations in pain sensitivity. Reduced pressure pain detection thresholds (PPTs) at the affected site may indicate the presence of peripheral sensitization, while reduced PPTs at a distal pain‐free side is suggestive of altered pain processing in the CNS and central pain augmentation (Croft et al., 2005; Graven‐Nielsen & Arendt‐Nielsen, 2002; Suokas et al., 2012). Magnetic resonance imaging‐detected inflammation in knee OA, and not the severity of radiographic features, is associated with the development and worsening of local pressure pain sensitivity in the knee (Neogi et al., 2016), suggesting that inflammation is a potential mechanism underlying local sensitization. Neuropathic pain symptoms on the painDETECT questionnaire have been associated with signs of central sensitization on QST, such as associations with PPTs at sites distant from the affected joints, suggesting that painDETECT may reflect central pain processing in patients with knee OA (Hochman et al., 2013; Moreton et al., 2015; Moss et al., 2018).

Genetic variants implicated in pain sensitivity have arisen from candidate gene studies. These included amino acid change variants in the catechol‐*O*‐methyltransferase (COMT) (van Meurs et al., 2009), the voltage‐gated sodium channel Nav1.7 (SCN9A) (Reimann et al., 2010) and the transient receptor potential cation channel, subfamily V, member 1 (TRPV1) (Valdes et al., 2011). However, there is a gap in our knowledge regarding the genes and molecular pathways influencing pain sensitization in knee OA. Evidence indicates that signs of increased pain sensitization might be a barrier to treatment response. Indeed, widespread hyperalgesia assessed by PPTs predicts poor outcomes to arthroplasty (Lundblad et al., 2008; Petersen et al., 2016; Rakel et al., 2012; Wylde et al., 2015). Furthermore, knee OA patients with neuropathic pain symptoms identified using the painDETECT questionnaire are most at risk of developing chronic postoperative pain after total knee replacement (Kurien et al., 2018). Therefore, understanding the underlying mechanisms involved in pain sensitization and neuropathic‐like pain may promote better profiling and diagnosis of pain patients and development of new regimes for mechanism‐based therapy. Thus, we completed two GWASs with PPTs from distal and affected sites to identify genetic variants for distal and local sensitization, respectively, using data from the Knee Pain and related health In the Community (KPIC) cohort (Fernandes et al., 2017). We then completed a genome‐wide gene‐based association analysis (GWGAS) and gene‐set analysis on both PPT phenotypes separately to explore the genes and underlying genetic mechanisms of distal and local sensitization in OA pain. Using existing GWAS data from an independent Nottingham cohort study of neuropathic‐like pain (Warner, Walsh, et al., 2017), we performed a GWGAS to identify genes related to the neuropathic‐like pain phenotype. GWGAS may have higher power to identify the causal variants of complex diseases compared to GWAS because it considers the joint effect of several single nucleotide polymorphisms within a single gene (Chung et al., 2019; Kang et al., 2013). As pain sensitization is suggested to display similar characteristics to neuropathic‐like pain, we performed two separate gene‐based meta‐analyses by combining our neuropathic‐like pain GWGAS findings with our findings from distal and local sensitization GWGAS to identify genes common in distal sensitization and neuropathic‐like pain, as well as local sensitization and neuropathic‐like pain, respectively.

## 2 METHODS

**FIGURE 1:**
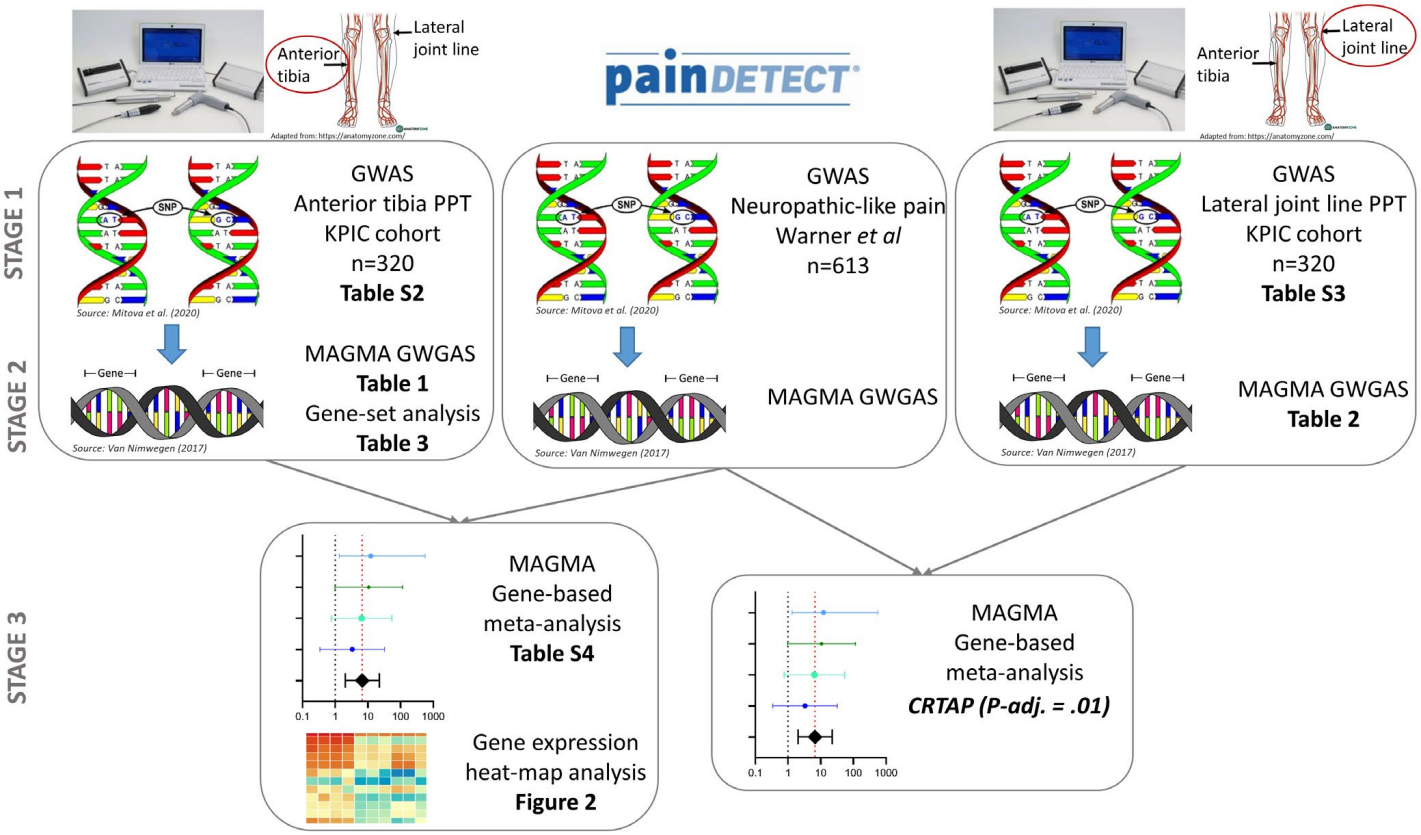
Study design. A three‐stage design for identification of associations between genetic variants and two pain sensitivity phenotypes: distal sensitization [anterior tibia pressure pain threshold (PPT)] and local sensitization (lateral joint line PPT), as well as genes common in pain sensitivity and neuropathic‐like pain (painDETECT questionnaire) phenotypes. First, two GWASs with PPTs from both distal and affected sites were run to identify genetic variants for distal and local sensitization, respectively, using data from the KPIC cohort (Tables S2 and S3). Second, three GWGASs and gene‐set analyses were run to identify the genes associated with the two pain sensitivity phenotypes and a neuropathic pain‐like phenotype using the GWASs outputs from KPIC and existing GWAS data from a Nottingham cohort (Tables 1, 2, 3). Third, two meta‐analyses of our GWGAS findings were conducted to identify genes common in pain sensitization (distal and local) and neuropathic‐like pain (Table S4) and a heat map analysis of the common genes identified from the distal sensitization/neuropathic‐like pain meta‐analysis was performed (Figure 2)

The study design is outlined in Figure 1. We used data from two independent study cohorts. Participants from both cohorts had similar demographic characteristics (age, sex and BMI). We used a three‐stage design for the identification of any potential associations between genetic variants and two pain sensitivity phenotypes (distal sensitization and local sensitization), as well as genes common in pain sensitivity and neuropathic‐like pain phenotypes. First, two GWASs with PPTs from both distal and affected sites were run to identify genetic variants for distal and local sensitization, respectively, using data from the KPIC cohort. Second, three GWGASs were run to identify the genes associated with the two pain sensitivity phenotypes and a neuropathic pain‐like phenotype using the GWASs outputs from KPIC and existing GWAS data from a Nottingham cohort. Third, we conducted two meta‐analyses of our GWGAS findings to identify genes common in pain sensitization (distal and local) and neuropathic‐like pain.

### 2.1 Participants

#### 2.1.1 KPIC cohort

Baseline data from a subgroup of the KPIC (*n* = 320) that undertook clinical assessments including knee radiographs, knee ultrasound, quantitative sensory testing, muscle strength, balance, gait analysis and biomarker sampling and for which we had genetic information and no missing data on anterior tibia and lateral joint line PPTs was used (Fernandes et al., 2017) (Table S1). KPIC is a prospective community‐based cohort of men and women aged 40 years or over within the East Midlands region (UK).

#### 2.1.2 Nottingham post‐total joint replacement (TJR) cohort

Participants were recruited post‐TJR for OA (*n* = 613) from secondary care in the Nottinghamshire area (Warner, Walsh, et al., 2017). Demographic characteristics of this cohort are presented in Table S1.

### 2.2 Phenotypes

#### 2.2.1 Neuropathic‐like pain

This phenotype has been described extensively elsewhere (Warner, van Meurs, et al., 2017). Briefly, neuropathic‐like pain was measured with the painDETECT (Freynhagen et al., 2006), a self‐report seven‐item questionnaire with scores ranging from 0 to 39 developed to discriminate between nociceptive and neuropathic pain. The questionnaire asks about the intensity, pattern and quality of their knee pain, for example persistent with slight fluctuations, burning, tingling or sudden, with Likert scale and tick‐box questions. Individuals were classified as having possible or probable neuropathic‐like pain if they scored >12 according to the validated cut‐offs for diagnosis by Freynhagen et al. (2006) making up 109 possible or probable neuropathic pain cases versus 504 controls (Warner, van Meurs, et al., 2017). The dichotomised value for the classification of participants to neuropathic‐like pain versus controls was used for the GWAS and the results from this analysis can be found elsewhere (Warner, van Meurs, et al., 2017).

#### 2.2.2 Pressure pain detection thresholds (PPT)

PPTs (in kPa) were measured in triplicate with an algometer (Somedic AB, Sweden) that is connected to a computer (HP ProBook 4520s). Pressure was administered manually at a progressively increasing rate (standardized rate set at 30 kPa/s) by a trained researcher through the algometer, a circular rubber‐coated pressure probe (1 cm^2^). Subjects were instructed to push a button when the sensation changed from pressure to pain and the algometer was immediately taken off the skin. Among other areas, PPT was applied to the lateral tibiofemoral joint line adjacent to the patellar ligament of the index knee and the anterior tibia 5 cm distal to the tibial tuberosity of the contralateral knee (Fernandes et al., 2017). PPT has been shown to be a reproducible measure of nerve sensitivity throughout localized, distal and remote sites and it is part of QST, which is used to quantify pain perception. PPT at sites away from the affected joint has been interpreted as an index of central sensitization (i.e. increased pain perception in areas away from the knee) (Arendt‐Nielsen et al., 2010; Pavlaković & Petzke, 2010). PPT at an affected joint may indicate a combination of peripheral and central sensitization. Results from a systematic review and meta‐analysis revealed that people with OA have lower PPTs both at the affected joint and at areas away from the joint when compared to controls (Suokas et al., 2012). The mean of PPT values for each site were computed from all three PPT rounds for analysis. Log transformation resulted in more symmetrical and less skewed distributions of the PPT values. Therefore, the log‐transformed variables of PPT values were used for all further analyses.

### 2.3 GWAS

For all cohort study participants, genomic DNA was extracted from peripheral blood leukocytes. Genotype data were analysed using the Illumina Global screening array Inc Basic BioIT (Illumina). Quality Control (QC) checks and genotyping were done both at the sample and single nucleotide polymorphism (SNP) level and have been described in detail elsewhere (Warner, Walsh, et al., 2017; Zeggini et al., 2012). PLINK software (version 1.07) was used to analyse GWAS data from this array (Purcell et al., 2007). Two GWASs were conducted to identify associated SNPs and genes with the two distal and local sensitization phenotypes, respectively. The GWAS output contains information about the SNPs’ location in the genome, a regression coefficient beta (β) and a test statistic that indicate the level of association of the genetic variants with the phenotype along with a *p* value to determine significance.

### 2.4 Functional annotation

To gain insights into the functions of the identified genes, we next tested the probability of these genes to map into specific biological pathways as defined by the Kyoto Encyclopaedia of Genes and Genomes (KEGG) or Reactome databases using the Database for Annotation, Visualisation and Integrated Discovery (DAVID) (Dennis et al., 2003) and Reactome online platforms, respectively. The gene list was composed of genes corresponding to all SNPs with a *p*‐value of *p* < 0.0001 in the GWAS analysis.

### 2.5 Gene‐based association analysis

Subsequently, our GWAS results were used for GWGAS using Multi‐marker Analysis of GenoMic Annotation (MAGMA) (de Leeuw et al., 2015). MAGMA takes as input the *p*‐values derived from the GWAS and annotates SNPs to known protein‐coding genes to estimate aggregate associations based on all SNPs in a gene (linkage disequilibrium) accounting for multi‐marker effects, by applying multiple regression analyses. MAGMA then uses Fisher's test to compute *p*‐values to test the association between a gene and the phenotype and does not assign a positive or negative coefficient to a gene‐based association. It differs from functional annotation as it provides a statistical gene‐based test, whereas functional annotation methods map individually significant SNPs to genes. The 1,000 Genomes Project (phase 1, release 3) was used as a reference panel to calculate linkage disequilibrium between genomic variants (Auton et al., 2015). A gene‐based analysis was performed for each phenotype using the results from our GWAS for local and distal sensitization and existing GWAS data for neuropathic‐like pain. The NCBI 37.3 build was used to obtain the SNPs that were attributed to each gene (de Leeuw et al., 2015). A total of 14,428, 14,428 and 14,722 protein‐coding genes were assessed for an association with the distal sensitization phenotype, the local sensitization phenotype and the neuropathic‐like pain phenotype, respectively. Benjamin–Hochberg (B–H) correction was used to determine significance.

### 2.6 Gene‐based meta‐analysis

Our GWGAS resulted in different genes for anterior tibia PPT and lateral joint line PPT. For anterior tibia PPT, we identified genes that have been previously related to central mechanisms of pain, whereas one of the lateral joint line PPT genes found, has been previously associated with peripheral pain mechanisms. Accordingly, we conducted two separate fixed‐effects gene meta‐analyses combining our GWGAS neuropathic‐like pain results with GWGAS results from anterior tibia PPT and lateral joint line PPT to identify genes common in neuropathic‐like pain and distal sensitization as well as neuropathic‐like pain and local sensitization, respectively. The *Z*‐scores for each gene across the two cohorts, KPIC and Nottingham post‐TJR, were combined using the weighted‐Z method. According to this method, the weights are computed as the inverse of the squared standard error of the effect size estimate for each cohort, resulting in different weights for each cohort according to their power (Lipták, 1958; Whitlock, 2005). B–H correction was used to determine significance.

### 2.7 Pathway over‐representation analysis

Significant results from the GWGAS were entered in FUMA for gene‐set/GWAS catalogue over‐representation analysis with MAGMA (de Leeuw et al., 2015; Watanabe et al., 2017). We identified statistically significant overrepresented pathways for the anterior tibia PPT phenotype after adjusting for false detection rate (FDR) with B–H correction method.

## 3 RESULTS

### 3.1 Stage 1: GWAS

The 10 ‘top‐hits’ (i.e. genes with highest *p*‐values that survived correction for multiple testing) of the adjusted GWAS on anterior tibia and lateral joint line PPT phenotypes are shown in Tables S2 and S3, respectively. The total genotyping rate was 0.99 with 700,078 variants passing filtering and QC and each tested for association with both phenotypes. The genomic inflation factor lambda (based on median chi‐square) was low for both phenotypes (λ = 1.07, for anterior tibia PPT and λ = 1.00, for lateral joint line PPT). After adjusting for multiple testing, 554,759 SNPs remained in the analysis. The results of GWAS on neuropathic‐like pain are described elsewhere (Warner, van Meurs, et al., 2017).

### 3.2 Stage 2: Gene‐based association analysis

As knee OA is a complex, polygenic disease, we next decided to perform gene‐based association analysis, which has been suggested to be more powerful at unravelling associations compared to GWAS, as it accounts for the correlations among SNPs within a single gene (Chung et al., 2019). The ‘top’ results, in terms of statistical significance (i.e. genes with highest *p*‐values that survived correction for multiple testing) as well as biological relevance, of the gene‐association analyses on anterior tibia and lateral tibiofemoral joint line PPT can be seen in Tables 1 and 2, respectively. Several of the genes identified for anterior tibia PPT phenotype are traditional pain‐related genes and members of the ‘sensory perception’ gene set according to the Gene Ontology definition (GO: 0007600) (i.e. *KCNA1*, *MTOR*, *ADORA1* and *SCN3B*). For lateral joint line PPT, we found a single gene, *PTAFR*, member of the ‘sensory perception’ gene set.

**TABLE 1:** The results of interest from the MAGMA gene‐based association analysis of anterior tibia PPT phenotype

Gene symbol	CHR.	Start position	Stop position	SNPS	*Z* statistic	Gene *p*‐value	Adjusted *p* *
OR5B3	11	58169938	58170882	1	9.48	1.22E‐21	1.75E‐17
WNT9A	1	228109165	228135676	1	8.59	4.36E‐18	3.14E‐14
DNAH7	6	29364416	29365448	2	7.04	9.47E‐13	2.48E‐09
IFNGR1	6	139456249	139501946	2	7.01	1.04E‐12	2.48E‐09
HECA	11	64863587	64879332	2	7.06	1.20E‐12	2.48E‐09
AIM2	1	159028790	159046685	3	6.98	1.48E‐12	2.68E‐09
POLR3E	16	22308696	22346424	3	5.69	6.41E‐09	4.87E‐06
IL10RA	11	117857106	117872198	3	5.33	4.95E‐08	2.98E‐05
HRH2	5	175084847	175136239	6	5.04	2.37E‐07	9.23E‐05
ROPN1	3	123687862	123711017	1	4.96	3.46E‐07	1.28E‐04
KCTD11	18	13218729	13652753	93	4.78	8.70E‐07	2.68E‐04
CCDC14	3	123616152	123680255	4	4.78	8.96E‐07	2.69E‐04
KCNA1	12	5019073	5027422	1	4.32	7.98E‐06	1.52E‐03
MTOR	1	11166588	11322614	6	3.98	3.40E‐05	4.42E‐03
HRH1	10	38383264	38412280	1	3.90	4.84E‐05	5.82E‐03
CACNB2	10	18429373	18830688	85	3.89	5.10E‐05	6.08E‐03
TNFAIP3	6	138663930	138790381	11	3.71	1.05E‐04	1.02E‐02
ADORA1	1	203096833	203136533	9	3.39	3.56E‐04	2.17E‐02
SCN3B	11	123499895	123525315	6	3.20	6.87E‐04	3.43E‐02
TAOK3	12	118587606	118810750	8	3.08	1.04E‐03	4.51E‐02
CACNA2D3	3	54156620	55108584	156	3.03	1.23E‐03	4.97E‐02

* Adjusted *p* values after applying B–H correction to control for multiple testing FDR.

**TABLE 2:** The results of interest from the MAGMA gene‐based association analysis of lateral joint line PPT phenotype

Gene symbol	CHR.	Start position	Stop position	SNPS	Z statistic	Gene *p*‐value	Adjusted *p* *
BRDT	1	92414928	92479985	4	6.92	2.24E‐12	3.23E‐08
RFX6	6	117198376	117253326	7	6.59	2.19E‐11	1.05E‐07
CRTAP	3	33155450	33189265	8	6.11	4.84E‐10	1.74E‐06
SUOX	12	56391043	56399309	2	6.06	6.73E‐10	1.94E‐06
CHD3	17	7788096	7816075	2	5.48	2.15E‐08	5.15E‐05
CRAT	9	131857073	131873070	2	5.18	1.10E‐07	1.97E‐04
IKZF4	12	56401268	56432219	5	4.92	4.31E‐07	6.90E‐04
TMEM26	10	63166401	63213208	5	4.36	6.53E‐06	8.95E‐03
IARS	9	94972489	95056038	1	4.35	6.83E‐06	8.95E‐03
PTAFR	1	28473677	28520447	1	4.02	2.89E‐05	2.78E‐02
C12ORF10	12	53693132	53700965	1	3.94	4.09E‐05	3.10E‐02
DAPK1	9	90112601	90323566	4	3.85	6.01E‐05	4.33E‐02

* Adjusted *p* values after applying B–H correction to control for multiple testing FDR.

We then performed a gene‐set analysis to identify pathways related to PPT phenotypes using FDR‐adjusted significant genes as identified from the GWGAS. We found significantly overrepresented pathways for anterior tibia PPT after B–H correction (Table 3). Enrichment was seen in several metabolic‐related pathways.

**TABLE 3:** Gene‐set analysis for anterior tibia PPT MAGMA FDR‐adjusted significant genes

GeneSet	*N*	*n*	*p*‐value	Adjusted *p*	Genes
Systolic blood pressure	793	26	1.88e‐7	2.52e‐4	CPSF3L, NME7, ADORA1, WNT9A, GPR137B, CACNB2, SYNPO2L, TCF7L2, NOX4, SYT1, MYCBP2, HOXB7, INSR, RGL3, SLC8A1, COBLL1, TNS1, COL4A4, JPH2, ITPR1, HRH1, FGD5, CTNNB1, MITF, ADRB2, TBXAS1
Obesity‐related traits	756	25	2.77e‐7	2.52e‐4	CSF1, AIM2, CACNB2, SLC29A3, NAV2, NOX4, IL10RA, ANO2, SYT1, FAM155A, SAMD4A, MAX, FAM189A1, CEP152, SLC8A1, MACROD2, ITPR1, NCEH1, TLL1, LRFN2, GRIK2, NXPH1, AUTS2, TBXAS1, HR
Night sleep phenotypes	538	20	7.48e‐7	4.51e‐4	SPTA1, WNT9A, LRIG3, SAMD4A, AEN, PMP22, ZNF830, RTTN, ZNF486, LRP1B, UBOX5, TASP1, DTD1, CACNA2D3, LRTM1, GALNTL6, NADK2, RANBP3L, EPB41L4A, HRH2
Amyotrophic lateral sclerosis (sporadic)	164	11	9.94e‐7	4.51e‐4	FANK1, RYR3, ANKRD29, RNF165, MACROD2, MYO18B, TAPT1, TLL1, LRFN2, AUTS2, TBXAS1
Coronary artery calcified atherosclerotic plaque score in type 2 diabetes	26	5	5.65e‐6	2.05e‐3	LRP1B, MAGI1, ZBTB49, OSBPL3, MTSS1
Systemic juvenile idiopathic arthritis	34	5	2.24e‐5	6.77e‐3	KLF17, WWOX, ZNF521, TAPT1, COL12A1
Body mass index	1365	31	2.65e‐5	6.88e‐3	MTOR, FAM63A, CACNB2, TCF7L2, PNLIPRP3, STK33, SFSWAP, POLR2 M, CHTF18, CYLD, MYO19, CDC27, POP4, LRP1B, COBLL1, COL4A4, MACROD2, ENTPD6, TAF4, CACNA2D3, COL25A1, FGF2, TLL1, LRFN2, COL19A1, IFNGR1, RGS17, SBDS, AUTS2, TSGA13, DMD
Hand grip strength	156	9	3.21e‐5	7.28e‐3	TCF7L2, SYT1, SAMD4A, SLC8A1, DNER, ITPR1, CADPS, LRFN2, AUTS2

Note

The results of pathway analysis showing overrepresented pathways, before and after B–H correction.

### 3.3 Stage 3: Gene‐based meta‐analysis

**FIGURE 2:**
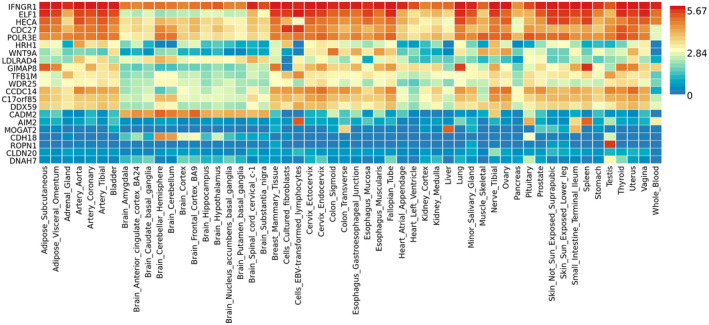
Gene expression heat‐map plot of significant genes from the meta‐analysis of anterior tibia PPT and neuropathic‐like pain in both cohorts

We then conducted a fixed‐effects gene meta‐analysis of anterior tibia PPT and neuropathic‐like pain in both cohorts (KPIC and Nottingham post‐TJR) and performed heat map analysis (Table S4 and Figure 2, respectively). Results from the meta‐analysis revealed ‘top‐hits’ (i.e. genes with highest *p*‐values that survived correction for multiple testing) in various genes related to bone morphogenesis and neuro‐inflammation, including *WNT9A*, *POLR3E*, *AIM2*, *HECA* and *IFNGR1* (Table S4). We visualized tissue specific expression patterns based on GTEx v6 RNA‐seq data (GTEx Consortium, 2015) for each significant gene as an interactive heat map plot (Figure 2).

We also performed a fixed‐effects gene meta‐analysis of lateral joint line PPT and neuropathic‐like pain in both cohorts, which yielded a single significant gene, *CRTAP* at chromosome 3 (*Z* = 4.83, *p*‐adjusted = 0.01, after applying B–H correction).

## 4 DISCUSSION

In this study, we used a GWGAS approach to identify novel genes for pain sensitization in knee OA. The most significant genes associated with distal and local sensitization were *OR5B3* and *BRDT*, respectively. Additionally, we identified several traditional pain‐related genes, *KCNA1*, *MTOR*, *ADORA1* and *SCN3B*, for anterior tibia PPT phenotype and *PTAFR* for lateral joint line PPT phenotype. Gene‐set analysis for the anterior tibia PPT revealed enrichment of metabolic‐related pathways. We found genes involved in bone morphogenesis (*CRTAP* and *WNT9A*), inflammation and neuroinflammation (*POLR3E*, *AIM2* and *IFNGR1*), metabolic disease and obesity (*AIM2*, *DNAH7*, *CADM2*), and cognitive function (*CRTAP* and *CCDC14*) from meta‐analysis of distal sensitization and neuropathic‐like pain traits in the two cohorts. A single gene, *CRTAP*, was significant after meta‐analysis of local sensitization and neuropathic‐like pain traits.

The most significant gene associated with distal sensitization was *OR5B3*. *OR5B3* is an olfactory receptor gene involved in the production of G‐protein coupled transmembrane receptors, which enable the detection and transmission of olfactory stimuli. A recent study revealed that knockdown of cathepsin S (CTSS) upregulates almost all olfactory receptor family genes and vice versa, in cell overexpressing CTSS and an increase of the expression level of olfactory receptor 5B3 protein was observed when cells were treated with a CTSS inhibitor (Chen et al., 2021). It has been shown that the release of CTSS from microglial cells causes neuropathic pain (Clark & Malcangio, 2012) that could be reversed by intraspinal injection of CTSS inhibitor. Consistent with our findings, these results suggest a potential mechanistic role of *OR5B3* in neuropathic pain. *BRDT* was the most significant gene associated with local sensitization and is a member of the bromodomain and extra‐terminal (BET) family of proteins. BET proteins bind to acetylated lysine residues in the histones of nucleosomal chromatin and function either as co‐activators or co‐repressors of gene expression (Filippakopoulos & Knapp, 2014), where they regulate the expression of key oncogenes, anti‐apoptotic proteins and many immunity‐associated genes and pathways (Wang et al., 2021), which is consistent with an underlying role of inflammation in local sensitization.

Genes previously associated with neuropathic pain and signs of central sensitization—*KCNA1*, *MTOR*, *ADORA1* and *SCN3B*—were identified from GWGAS of anterior tibia PPT, while an inflammatory pain gene—*PTAFR*—was found from GWGAS of lateral joint line. These findings are consistent with a role of joint damage and inflammation in local sensitization, as well as altered pain processing in the CNS in distal sensitization, and thus are biologically plausible. Specifically, *KCNA1* encodes for the potassium voltage‐gated channel subfamily A member 1, which is a key player in the perception of mechanical stimuli (Hao et al., 2013). *ADORA1* that codes for adenosine A_1_ receptor is suggested to have anti‐nociceptive properties, following surgery (Gan & Habib, 2007), and to reduce thermal hyperalgesia and mechanical allodynia in animal models of neuropathic pain (Gong et al., 2010; Wu et al., 2005). *MTOR* that codes for the mammalian target of rapamycin has a role in inflammatory‐ and opioid‐induced hyperalgesia (Xu et al., 2011, 2014) and acts as a regulator of neuroplasticity in the CNS (Hoeffer & Klann, 2010; Jaworski & Sheng, 2006). *SCN3B* encodes for the b3 subunit of voltage‐gated sodium channel (Na_v_) (Morgan et al., 2000) and is implicated in neuropathic pain (Casula et al., 2004; Lopez‐Santiago et al., 2006; Pertin et al., 2005). Finally, *PTAFR* encodes for a member of the G‐protein coupled receptor 1 family of proteins for platelet‐activating factor and is involved in the perception and maintenance of neuropathic pain by regulating the production of pro‐inflammatory cytokines in the dorsal root ganglion (Okubo et al., 2012; Shindou et al., 2017; Tsuda et al., 2011).

The results of pathway analysis for anterior tibia PPT revealed overrepresented pathways associated with systolic blood pressure, obesity‐related traits, night sleep phenotypes, amyotrophic lateral sclerosis, type 2 diabetes, systemic juvenile idiopathic arthritis, body mass index and hand grip strength. These traits are often comorbid with OA and have been previously associated with pain from OA (Swain et al., 2020). For instance, cardiovascular disease and OA share similar underlying disease mechanisms (Fernandes & Valdes, 2015). Obesity is a major risk factor for OA and shares common genetic variations with OA (Panoutsopoulou et al., 2014). Disturbed sleep has been associated with increased pain in OA (Doherty & Smith, 1993; Smith et al., 2009), while pain has been suggested to increase the risk of developing frailty (low hand grip strength) in OA (Valdes & Stocks, 2018; Veronese et al., 2017).

Meta‐analysis of distal sensitization and neuropathic‐like pain revealed ‘top‐hits’ in genes involved in bone morphogenesis, *CRTAP* (Morello et al., 2006) and *WNT9A* (Regard et al., 2012). A recent GWAS identified *WNT9A* as a robust novel genetic marker for hand OA (Boer et al., 2021). We identified several inflammation‐related genes, *POLR3E* (Chiu et al., 2009), *AIM2* (Hornung et al., 2009), *IFNGR1* (van de Wetering et al., 2010), *LDLRAD4* (Nakano et al., 2014) and *HRH1* (Dong et al., 2014). Increased accumulation of pro‐inflammatory factors in the joint accompanying synovitis, is highly correlated to OA pain (Berenbaum, 2013). Despite the role of *POLR3E*, *AIM2*, *IFNGR1* in inflammation, evidence suggests that these genes are also involved in neuroinflammation, neurodegeneration and neuroplasticity, processes related to central pain facilitation and neuropathic pain (Ji et al., 2018; Latremoliere & Woolf, 2009; Myers et al., 2006). For example, *POLR3E* participates in pre‐mRNA splicing and transcription and aged mice showed reduced expression of *POLR3E* (Kohman et al., 2011). Improper RNA splicing can result in abnormal translation of RNA and is associated with many age‐related diseases including Alzheimer's disease (Meshorer & Soreq, 2002). Furthermore, *AIM2* is involved in neuroinflammation and neurodegeneration (Cox et al., 2015; Wu et al., 2017), as well as in neuronal plasticity and memory (Chen et al., 2019; Wu et al., 2016). Moreover, deletion of *IFNGR1* results in complete abrogation of neuroinflammation and nigrostriatal degeneration, suggesting a role of this gene in neuroinflammation and neurodegeneration (Strickland et al., 2018). We also identified genes involved in obesity and adiposity, *AIM2* (Gong et al., 2019), *IFNGR1* (Locke et al., 2015), *DNAH7* (Söhle et al., 2012) and *CADM2* (Graff et al., 2017), as well as type 2 diabetes and cardiovascular disease, *DNAH7* (Vujkovic et al., 2020), *DDX59* (van der Harst & Verweij, 2018), *WDR25* (Perry et al., 2014) and *CLDN20* (Heid et al., 2010). Additionally, we found genes associated with intelligence, educational attainment, cognitive performance and psychological traits, *CRTAP* (Hall et al., 2018), *CCDC14* (Hill et al., 2019), *TFB1 M*, *ROPN1* (Lee et al., 2018), *HRH1* (Shan et al., 2015) and *CADM2* (Okbay et al., 2016). There is strong epidemiological evidence of a link between cognitive function, depression/anxiety and pain in people with arthritis (James & Ferguson, 2019).

We do not find significant associations with some of the candidate genes previously reported to be associated with a pain phenotype, such as *COMT*, *SCN9A* and *TRPV1*, in our GWGAS. This can be partly explained due to different analysis methods. In candidate gene studies, genes are selected *a priori* and only very small regions of the genome are investigated at a time, meaning that important genes may be overlooked using this method (Warner & Valdes, 2017). Furthermore, a lack of reproducibility of SNPs in candidate genes in GWAS meta‐analyses has been previously shown. For example, candidate *COMT* SNPs were not reproduced in a GWAS meta‐analysis of chronic widespread pain (Peters et al., 2013). In addition, although *COMT* has been extensively studied in relation to pain, results from candidate gene studies are not consistent (Hagen et al., 2006; Hocking et al., 2010; Nicholl et al., 2010). Rare and drastic mutations in the *SCN9A* gene that explain different types of congenital insensitivity to pain have been identified (Cox et al., 2006). Nevertheless, in addition to these rare‐causing mutations, it is known that the genetic risk for chronic pain is due to common variations with small effect size (Mogil, 2012). *TRPV1* has been associated with heat pain sensitivity, and thus it is not necessarily implicated in mechanical pain transduction. Indeed, it was shown that TrpV1 neurons are selectively tuned nociceptors that mediate responses to thermal but not mechanical pain (Mishra & Hoon, 2010).

Limitations of the current study include the small sample sizes of the individual cohorts used that are not sufficient to power genome‐wide significant results. We tried to overcome this by running gene‐based meta‐analysis of the two cohorts. Findings from GWAS are generally prone to type 1 errors (Bacanu et al., 2000). This was addressed by adjusting results for FDR with B–H (Benjamini, 2010). Another limitation with the use of GWAS is the possibility of inflated effect sizes (Ioannidis, 2008). We performed GWGAS and gene‐set analysis that may have higher power to identify the causal variants of complex diseases, as it takes into consideration the correlations among SNPs within a single gene (Kang et al., 2013). Gene‐based tests are designed to identify genes containing multiple risk variants that individually are weakly associated with a univariate trait (Chung et al., 2019). There is a possibility that distal PPTs are reflective of central sensitization mechanisms but can also reflect features of peripheral (inflammatory) mechanisms and while neuropathic pain is presumed to result from abnormal neuronal activity of the somatosensory nervous system, glial cell dysfunction may also contribute (Ji et al., 2013). Indeed, distal and local PPTs were correlated in our cohort (*r* = 0.72, *p* < 0.001) and from our meta‐analysis of distal sensitization and neuropathic‐like pain, we found some inflammation‐related genes. However, these genes are also associated with neuroinflammation, neurodegeneration and neuroplasticity, processes related to central pain processing and neuropathic‐like pain (Ji et al., 2018; Latremoliere & Woolf, 2009; Myers et al., 2006). In addition, painDETECT scores apart from being associated with signs of central sensitization (e.g. reduced PPTs) (Hochman et al., 2013) can also reflect nerve damage (Sumitani et al., 2016). Although the majority of the patients in the KPIC cohort had unilateral OA (75.19%), 33 patients had bilateral OA and thus, for these patients the contralateral tibia could be an OA affected area.

To conclude, our results suggest that different biological processes might be involved in distal and local sensitization, while common genetic mechanisms might be implicated in distal sensitization and neuropathic‐like pain. Further research is needed to confirm these findings and to explore whether other measures of altered pain processing (temporal summation and conditioned pain modulation) demonstrate similar results.

## CONFLICTS OF INTEREST

None to declare.

## AUTHOR CONTRIBUTIONS

AK is the primary author and all other authors are secondary. AMV is the main supervisor and is leading this project. All authors discussed the results and commented on the manuscript.

## Supporting information

Table S1‐S4
