# Supplementary material for: Different genes may be involved in distal and local sensitization: A genome‐wide gene‐based association study and meta‐analysis
Source: Eur J Pain. 2022 Jan 7;26(3):740–53. doi: 10.1002/ejp.1902 (PMC9303629; doi:10.1002/ejp.1902)
Supplement: Supplementary file 1 — Table S1‐S4 [file EJP-26-740-s001.docx]

**Table S1.** Demographic characteristics of the KPIC subgroup and the Nottingham post-TJR cohort.

| **Characteristics** | **KPIC**  **(n=320)^1^** | **Radiographic knee OA (n=133)^1^** | **Controls (n=187)^1^** | **Nottingham post-TJR**  **(n=613)^2^** | **Neuropathic-like pain**  **(n=109)^2^** | **Controls**  **(n=504)^2^** |
| --- | --- | --- | --- | --- | --- | --- |
| Age | 60.66 (9.40) | 63.04 (8.78) | 58.96 (9.48) | 70.98 (7.01) | 69.79 (7.34) | 71.35 (6.87) |
| Sex (%, female) | 60.63 | 41.24 | 58.76 | 54.16 | 63.19 | 51.28 |
| BMI | 28.80 (5.79) | 30.06 (6.49) | 27.89 (5.05) | 30.44 (5.40) | 31.50 (5.78) | 30.12 (5.24) |
| Knee pain* | 4.67 (3.35) | 6.71 (2.49) | 3.21 (3.13) | 4.15 (3.69) | 7.85 (1.77) | 3.02 (3.37) |
| Opioids (%) | 12.78 | 22.56 | 8.56 | 30.69 | 38.50 | 28.99 |
| Anti-neuropathics (%) | 3.75 | 6.77 | 1.60 | 6.12 | 11.00 | 5.06 |
| Anxiety**  % high/moderate | 6.71 (4.32)  17.18 | 7.76 (4.51)  24.81 | 5.96 (4.03)  11.76 | - | - | - |
| Depression**  % high/moderate | 4.78 (3.53)  7.81 | 5.76 (3.73)  9.77 | 4.08 (3.22)  6.42 | -  19.01 | -  29.86 | -  16.67 |

*^1^The KPIC cohort includes 320 participants—133 individuals with radiographic knee OA (Kellgren–Lawrence radiographic score >2 in the tibiofemoral or patellofemoral compartments of either knee) and current knee pain (present most days, lasting more than 3 months), plus 187 controls (i.e. without radiographic knee OA and without persistent, lasting pain). For the KPIC cohort, no binary classification was carried out, pressure pain thresholds were analysed as a continuous variable adjusting for radiographic knee OA status.*

*^2^The Nottingham post-TJR cohort includes 613 individuals with severe OA who had undergone a joint replacement and pain where present refers to persistent joint pain after one or more years of surgery. For this cohort, individuals were classified as having possible or probable neuropathic-like pain (i.e. PainDETECT score > 12 on a scale from 0 to 39) or not (lower PainDETECT scores) making up 109 neuropathic-like pain participants versus 504 controls.* *The dichotomised value for the classification of participants to neuropathic-like pain versus controls was used for analysis and the results can be found in Warner, van Meurs, et al. (2017).*

**Knee pain was measured with the Visual Analogue Scale*

**Anxiety and Depression were assessed with the Hospital Anxiety and Depression scale (HADS). Depression and anxiety were categorised using established clinical cutoffs for this instrument: moderate/high if HADS score > 10 and absent/low if HADS ≤ 10. Anxiety was not measured in the Nottingham cohort and thus is not reported.

**Table S2.** The results of the top 10 SNPs from the adjusted Illumina array anterior tibia PPT GWAS and their corresponding genes

| SNP ID | Location | CHR | BP | Effect  allele | Gene or closest gene | Beta | STAT | GWAS P | Adjusted P* |
| --- | --- | --- | --- | --- | --- | --- | --- | --- | --- |
| rs140673111 | 17:69194427-69194427 | 17 | 67190568 | G | ABCA10 | -2.56 | -10.42 | 5.22E-22 | 2.86E-18 |
| rs2099135 | 8:111438263-111438263 | 8 | 112450492 | G | LINC02237 | -2.56 | -10.34 | 1.01E-21 | 2.86E-18 |
| rs113274983 | 10:44876147-44876147 | 10 | 45371595 | G | TMEM72-AS1 | -2.56 | -10.33 | 1.02E-21 | 2.86E-18 |
| rs79567074 | 19:390200-390200 | 19 | 390200 | A | AC010641.2 | -2.56 | -10.32 | 1.12E-21 | 2.86E-18 |
| rs77959805 | 12:14893636-14893636 | 12 | 15046570 | G | C12orf60 | -2.56 | -10.31 | 1.18E-21 | 2.86E-18 |
| rs71524349 | 4:6289024-6289024 | 4 | 6290751 | C | WFS1 | -2.56 | -10.31 | 1.19E-21 | 2.86E-18 |
| rs3805048 | X:32488618-32488618 | 23 | 32506735 | C | DMD | -1.28 | -10.31 | 1.21E-21 | 2.86E-18 |
| rs112247130 | 6:44301434-44301434 | 6 | 44269171 | A | AARS2 | -2.56 | -10.3 | 1.21E-21 | 2.86E-18 |
| rs112942946 | 18:39220166-39220166 | 18 | 36800130 | A | MIR924HG | -2.56 | -10.3 | 1.21E-21 | 2.86E-18 |
| rs113993320 | 4:99420661-99420661 | 4 | 100341818 | G | ADH7 | -2.56 | -10.3 | 1.21E-21 | 2.86E-18 |

*adjusted P value after correction for multiple testing with B–H

**Table S3**.The results of the top 10 SNPs from the adjusted Illumina array lateral joint line PPT GWAS and their corresponding genes

| SNP ID | Location | CHR | BP | Effect  allele | Gene or closest gene | Beta | STAT | GWAS P | Adjusted P* |
| --- | --- | --- | --- | --- | --- | --- | --- | --- | --- |
| rs146956711 | 18:75287913-75287913 | 18 | 72999868 | A | TSHZ1 | -2.75 | -10.96 | 6.88E-24 | 2.25E-19 |
| rs201446493 | 6:116925456-116925456 | 6 | 117246619 | A | RFX6 | -2.75 | -10.96 | 6.88E-24 | 2.25E-19 |
| rs112246679 | 1:91976319-91976319 | 1 | 92441876 | A | BRDT | -2.75 | -10.96 | 6.88E-24 | 2.25E-19 |
| rs6128548 | 20:59363367-59363367 | 20 | 57938422 | A | AL035250.2 | -2.75 | -10.96 | 6.88E-24 | 2.25E-19 |
| rs116877798 | 13:98853444-98853444 | 13 | 99505698 | G | DOCK9 | -2.75 | -10.96 | 6.88E-24 | 2.25E-19 |
| rs76603930 | 3:38059757-38059757 | 3 | 38101248 | A | DLEC1 | -2.75 | -10.96 | 6.88E-24 | 2.25E-19 |
| rs72839866 | 17:6018700-6018700 | 17 | 5922020 | A | AC007846.2 | -2.75 | -10.96 | 6.88E-24 | 2.25E-19 |
| rs115198029 | 3:33132671-33132671 | 3 | 33174163 | A | CRTAP | -2.75 | -10.94 | 7.93E-24 | 2.32E-19 |
| rs145320435 | 14:60125773-60125773 | 14 | 60592491 | G | PCNX4 | -1.71 | -9.195 | 5.32E-18 | 1.34E-13 |
| rs75600413 | 12:56004018-56004018 | 12 | 56415348 | A | IKZF4 | -1.51 | -7.945 | 3.47E-14 | 7.71E-10 |

*adjusted P value after correction for multiple testing with B–H

**Table S4.** The results from the fixed-effects gene meta-analysis of anterior tibia PPT (KPIC cohort) and neuropathic-like pain (Nottingham post-TJR cohort).

| Gene name | CHR | ZSTAT | P | FDR-BH P |
| --- | --- | --- | --- | --- |
| Wnt family member 9A(WNT9A) | 1 | 5.71 | 5.79E-09 | 9.83E-05 |
| RNA polymerase III subunit E(POLR3E) | 16 | 5.25 | 7.58E-08 | 4.39E-04 |
| absent in melanoma 2(AIM2) | 1 | 5.25 | 7.75E-08 | 4.39E-04 |
| hdc homolog, cell cycle regulator(HECA) | 6 | 5.03 | 2.49E-07 | 1.06E-03 |
| interferon gamma receptor 1(IFNGR1) | 6 | 4.95 | 3.67E-07 | 1.06E-03 |
| dynein axonemal heavy chain 7(DNAH7) | 2 | 4.95 | 3.74E-07 | 1.06E-03 |
| coiled-coil domain containing 14(CCDC14) | 3 | 4.80 | 8.04E-07 | 1.95E-03 |
| transcription factor B1, mitochondrial(TFB1M) | 6 | 4.48 | 3.67E-06 | 7.71E-03 |
| cell division cycle 27(CDC27) | 17 | 4.45 | 4.24E-06 | 7.71E-03 |
| DEAD-box helicase 59(DDX59) | 1 | 4.43 | 4.70E-06 | 7.71E-03 |
| WD repeat domain 25(WDR25) | 14 | 4.42 | 4.99E-06 | 7.71E-03 |
| rhophilin associated tail protein 1(ROPN1) | 3 | 4.38 | 5.92E-06 | 8.38E-03 |
| low density lipoprotein receptor class A domain containing 4(LDLRAD4) | 18 | 4.33 | 7.57E-06 | 9.89E-03 |
| claudin 20(CLDN20) | 6 | 4.22 | 1.23E-05 | 1.48E-02 |
| histamine receptor H1(HRH1) | 3 | 4.20 | 1.31E-05 | 1.48E-02 |
| E74 like ETS transcription factor 1(ELF1) | 13 | 4.16 | 1.59E-05 | 1.69E-02 |
| cell adhesion molecule 2(CADM2) | 3 | 4.11 | 1.94E-05 | 1.94E-02 |
| nuclear cap binding subunit 3(NCBP3) | 17 | 4.06 | 2.46E-05 | 2.33E-02 |
| GTPase, IMAP family member 8(GIMAP8) | 7 | 4.03 | 2.77E-05 | 2.48E-02 |
| monoacylglycerol O-acyltransferase 2(MOGAT2) | 11 | 3.97 | 3.56E-05 | 3.02E-02 |
| cadherin 18(CDH18) | 5 | 3.92 | 4.43E-05 | 3.58E-02 |

*adjusted P value after correction for multiple testing with B–H
